# Elongation vs stalling: place your BET

**DOI:** 10.18632/oncotarget.22989

**Published:** 2017-12-06

**Authors:** Elisa Donato, Ottavio Croci, Stefano Campaner

**Affiliations:** Stefano Campaner: Center for Genomic Science, CGS@SEMM, Fondazione Istituto Italiano di Tecnologia (IIT), Milan, Italy

**Keywords:** BRD4, BET inhibitors, JQ1, CDK9

In the past years, there has been a growing attention towards crafting personalized cancer therapies based on epigenetic targets. In particular, the field made a major leap forward triggered by the possibility to pharmacologically target the activity of BRD4, a chromatin reader of the BET family of bromodomain proteins. This remarkable interest stemmed from the early promises held by inhibitors like JQ1 and I-BET151, which showed strong therapeutic efficacy in selectively inhibiting the expression of cellular oncogenes [1-4]. This was quickly reinforced by the mounting reports describing broad preclinical efficacy of these inhibitors in a variety of cancers.

BRD4 is a chromatin reader characterized by the presence of two functional bromodomains located at the N-terminus of the protein and one extra-terminal domain. The two bromodomains have a preferential binding activity for acetylated histone tails (in particular histones H3 and H4) and mediate BRD4 binding to chromatin. Previous studies had identified BRD4 as a regulator of transcription which, by favoring the activation of the pTEFb complex (CycT1/Cdk9), was able to promote RNApol2 elongation (Yang, Z. et al. Mol Cell, 2005. 19(4): 535-45).

Genome-wide chromatin immuno-precipitation (ChIP-seq) revealed broad genome interaction with a clear preferential binding of BRD4 to regulatory regions, both promoters and enhancers, bearing acetylated histones. Overall, this suggested that BRD4 may promote transcription by regulating activity and recruitment of the pTEFb complex to link distal regulatory elements (i.e. enhancers) to promoters.

A first mechanism accounting for the therapeutic efficacy of BET inhibitors relies on direct transcriptional inhibition of driver oncogenes. The initial evidence came from the studies on hematological malignancies, such as multiple myelomas, which are characterized by recurrent chromosomal rearragements involving the enhancers of the IgH locus and the c-Myc cellular oncogene [2]. In these translocations, the IgH enhancers are heavily acetylated and bound by BRD4. Pharmacological eviction of BRD4 from these enhancers inhibited the expression of the Myc oncogene, suggesting a key role for BRD4 in maintaining the regulatory activity of such enhancers. This mode of action was later generalized thanks to the identification of broad regulatory regions, named Super-Enhancers (SEs), which span several megabases and encompass numerous highly acetylated enhancers. SEs function as regulatory hubs of gene transcription by controlling the expression of both master regulators of cell identity and cellular oncogenes. These regions are avidly bound by BRD4 and could be selectively decommissioned by BET inhibitors [5, 6]. While these initial reports emphasized the role of BRD4 in integrating transcriptional responses dictated by distal regulatory elements, later on there has been growing awareness that BRD4 plays also a relevant role in regulating gene activity at promoters [7]. These data are in accordance with the original reports describing BRD4 as a positive regulator of elongation and are reinforced by ChIP-seq data which show pervasive promoter associated binding of BRD4 which scales with the enrichments of both RNApol2 and transcription factors and correlates with robust gene activity [8]. Importantly, while virtually all transcribed genes are bound and regulated by BRD4, expression of these genes is not equally affected by tonic inhibition of BRD4 [8].

The molecular bases of this apparent paradox lie in the identification of the rate-limiting event in mRNA synthesis. There are two fundamental steps that determine the overall efficiency (processivity) of the RNApol2 enzyme, the first is initiation: as soon as RNApol2 is recruited to promoters, it gets phosphorylated on Ser5 of its C-terminal domain (CTD), starts elongating with limited efficiency and pauses shortly downstream the transcriptional start site. Productive transcription involves the engagement of a second step, elongation, whereby further phosphorylation of the CTD on Ser2 (catalyzed by the pTEFb complex), promotes RNApol2 pause-release and its full activation. While the vast majority of transcribed genes elongate in a BRD4 dependent fashion, their sensitivity to tonic BRD4 inhibition depends on their transcriptional flux. Highly transcribed genes have maximal promoter recruitment (promoter associated RNApol2 is at the equilibrium, i.e. recruitment is more efficient than elongation) and strong elongation rates. On the other hand, moderately expressed genes rely on the balance between efficiency of RNApol2 recruitment and elongation (recruitment and elongation have comparable efficiency/rate).

These two different scenarios have a deep impact on how these two classes of genes will respond to BRD4 inhibition: while acutely (i.e. short term) BRD4 inhibition will affect elongation of both classes of genes thus causing a global decrease in mRNA synthesis, upon prolonged treatment adaptive responses will shape tonic transcription leading to selective downregulation of highly expressed genes [8].

This because low/moderately expressed genes will re-adjust to the drop in transcription by increasing the recruitment of RNApol2 on their promoters, thus reaching a new steady state; here increased promoter-paused RNApol2 compensates for decreased elongation and allows steady mRNA synthesis (this is based on the following: the amount of elongating RNApol2 = [Pol2^stalled^] x K^elongation^, where K is the elongation rate. If K decreases and stalled-Pol2 increases then elongation is buffered). Conversely, highly expressed genes, which in order to support robust transcription have maximized RNApol2 recruitment, are kinetically limited by elongation and thus are unable to mount such compensatory responses.

This implies an inherent balancing mechanism that on the one hand provides transcriptional buffering capabilities to moderately expressed genes but on the other poses an intrinsic liability on highly transcribed genes. As a corollary, this may explain the selective sensitivity of cancer cells to general transcriptional inhibitors, since these cells, by being addicted to high rates of mRNA synthesis, would be impaired in mounting compensatory responses. This mechanism of transcriptional buffering provides a rationale for the evaluation of inhibitors of transcription as selective therapeutic agents and highlights the need for further deepening our understanding on transcription as an Achille’s heel of tumor cells.
